# Carbon ion therapy for ameloblastic carcinoma

**DOI:** 10.1186/1748-717X-6-13

**Published:** 2011-02-06

**Authors:** Alexandra D Jensen, Swantje Ecker, Malte Ellerbrock, Anna Nikoghosyan, Jürgen Debus, Marc W Münter

**Affiliations:** 1 Dept of Radiation Oncology, INF 400, 69120 Heidelberg, Germany; 2 Dept. of Medical Physics, Heidelberg Ion Therapy Centre (HIT), INF 450, 69120 Heidelberg, Germany

## Abstract

Ameloblastic carcinomas are rare odontogenic tumors. Treatment usually consists of surgical resection and sometimes adjuvant radiation. We report the case of a 71 year-old male patient undergoing carbon ion therapy for extensive local relapse of ameloblastic carcinoma. Treatment outcome was favourable with a complete remission at 6 weeks post completion of radiotherapy while RT-treatment itself was tolerated well with only mild side effects. High dose radiation hence is a potential alternative for patients unfit or unwilling to undergo extensive surgery or in cases when only a subtotal resection is planned or the resection is mutilating.

## Introduction

Ameloblastic carcinomas are very rare odontogenic tumors sometimes referred to as malignant ameloblastoma [1]. To date, 31 cases have been reported worldwide, with the largest series describing 14 cases [2]

Definitions and classifications of ameloblastic carcinomas have changed over the years, there have been various classifications, the latest by Slootweg and Müller [3] emphasizing histogenesis of the tumor leading to the new WHO classification in 2005 [4]. Slootweg and Müller defined ameloblastic carcinoma as a tumor combining morphologic features of both ameloblastoma and carcinoma, which can arise de novo, ex ameloblastoma, or ex odontogenic cyst [3].

Most of the reported cases were discovered in the mandible, only one fifth occur in the maxilla [5,6]. Though they claimed to exhibit a tendency towards aggressive local growth and local relapse [1], distant metastases are uncommon. Being a rare disease, there are no treatment guidelines. However, standard treatment has been complete surgical resection in reported cases [5,7].

## Report of a case

**Figure 1:**
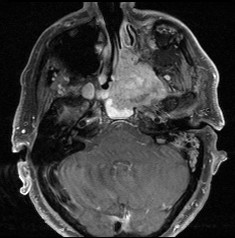
**Extensive ameloblastic carcinoma originating from the left maxilla: axial, contrast-enhanced T1 weighted MRI**.

**Figure 2:**
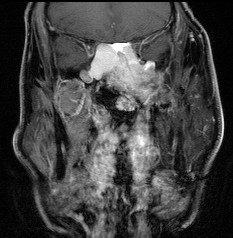
**Extensive ameloblastic carcinoma originating from the left maxilla: coronal, contrast-enhanced T1 weighted MRI**.

A 71-year-old patient with extensive relapse of an ameloblastic carcinoma was referred to our institution for carbon ion therapy by his maxillo-facial surgeon. He had been initially diagnosed 10 years ago when the tumor was completely resected. Local recurrence was found on a follow-up MRI scan 6 months prior to presentation in our department with the patient complaining of increasing pain in the left maxilla and probe excision confirming the diagnosis of locally recurrent ameloblastic carcinoma of the maxilla (Figures 1 and 2). As the referring surgeon doubted successful removal of the extensive tumor mass, faced with the inherent morbidity, salvage surgery did not seem a feasible option. Pre-therapeutic staging did not show signs of distant spread and the patient was referred to radiotherapy.

Interdisciplinary discussion recommended high-dose carbon ion therapy for this patient, hence he received a dose of 60 GyE carbon ions.

## Radiotherapy

### Immobilization/planning examinations

Immobilization consisted of an individual thermoplastic head mask with thermoplastic shoulder fixation. Planning examinations consisted of a planning CT scan (3 mm slice thickness) with the patient positioned in the individual fixation device and contrast-enhanced MRI for 3 D image correlation.

### Target volumes/dose prescription

CTV1 included the macroscopic tumor. PTV1 consisted of a 3 mm margin around the CTV1 but did not extend into critical organs at risk (i.e. brain stem, spinal cord). CTV2 included CTV1 and all involved paranasal sinuses. As there were no suspect lymph nodes, elective nodal irradiation was not performed given the rarity of nodal metastases.

**Figure 3:**
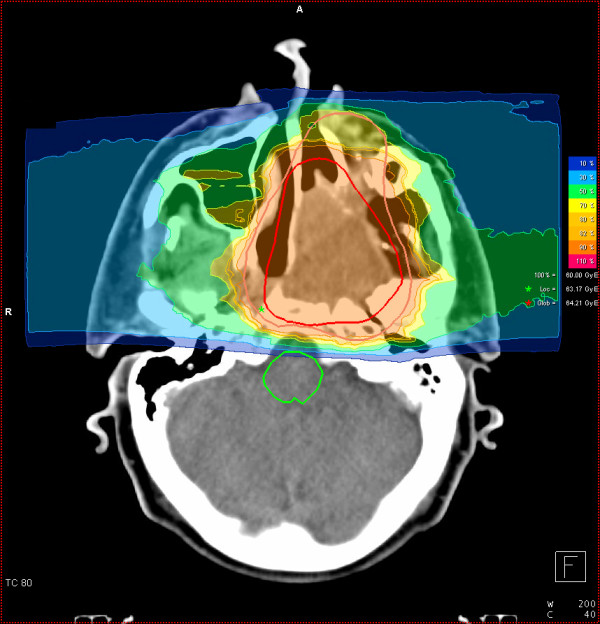
**Carbon ion dose distribution: (summation: primary plan to 45 GyE C12, boost plan to 15 GyE) 100% corresponding to 60 GyE, axial view**. Orange outline: CTV2; red outline: CTV 1; Green star: local maximum dose within the slice. Red star: global maximum.

**Figure 4:**
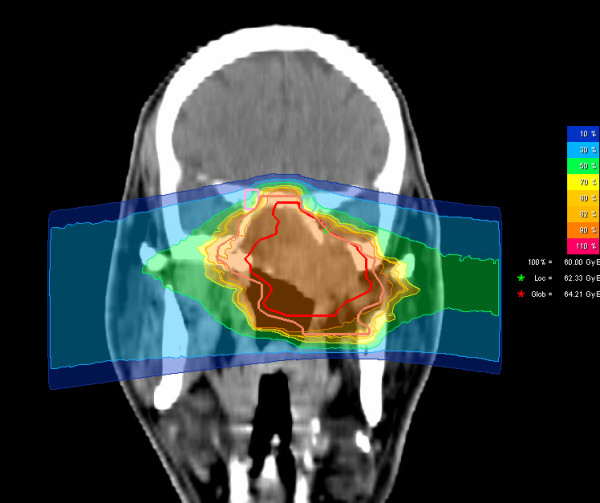
**Carbon ion dose distribution: (summation: primary plan to 45 GyE C12, boost plan to 15 GyE) 100% corresponding to 60 GyE, coronal view**. Orange outline: CTV2; red outline: CTV 1; Green star: local maximum dose within the slice. Red star: global maximum.

**Figure 5:**
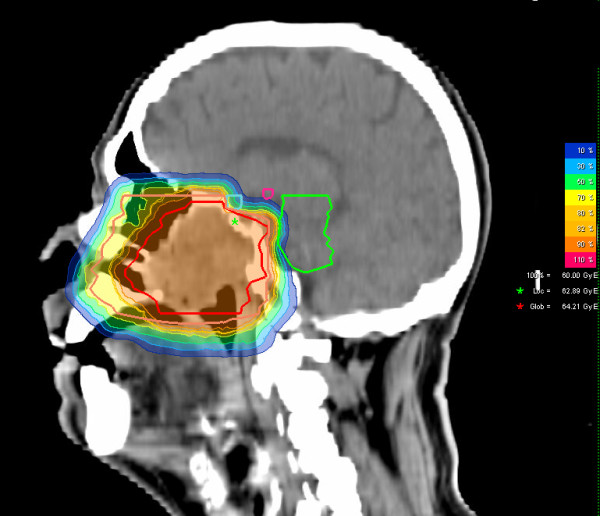
**Carbon ion dose distribution: (summation: primary plan to 45 GyE C12, boost plan to 15 GyE) 100% corresponding to 60 GyE, sagittal view**. Orange outline: CTV2; red outline: CTV 1; Green star: local maximum dose within the slice. Red star: global maximum.

**Figure 6:**
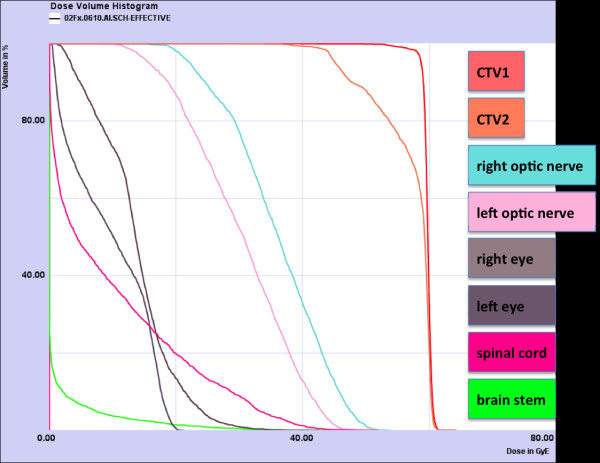
**Carbon ion summation plan: DVH**.

We prescribed a dose of 44,8 GyE carbon ions in 2,99 GyE/fraction (5 fractions per week) to the CTV2, followed by a boost to CTV1 with 14,9 GyE at 2,98 GyE/fraction. aiming at covering the CTV with the 95% prescription isodose. Figures 3, 4, and 5 show axial, coronal, and sagittal views of the summation dose distribution (voxel-by-voxel addition of basic and boost plan) with 100% corresponding to 60.0 GyE. Figure 6 depicts the dose-volume-histogram (DVH). Treatment was given at the HIT (Heidelberg Ion-Beam Therapy Centre) after inverse treatment planning in active beam application (raster-scanning method) [8]. A monoenergetic carbon ion beam with a full-width/half-maximum (FWHM) of 5 mm is extracted from the accelerator system (synchrotron) and magnetically deflected to subsequently scan all planned iso-energetic slices roughly corresponding to the tumour's radiological depth. Using this method almost any desired dose distribution can be created and dose to surrounding critical structures can be minimized.

Daily image guidance consisted of orthogonal x-ray controls in treatment position with the x-ray tube/receptor mounted on a robot to allow imaging in almost any treatment table position. After acquisition of orthogonal x-rays, an automatic 2D-3 D pre-match was carried out (Siemens syngo PT treatment) and verified by the radiotherapist/radiation oncologist with regard to bony anatomy. Manual adjustment of the match was carried out on-line and the resulting correction vector, including rotations, subsequently applied to the patient position. Patient position was controlled in each session and shifts were always corrected using a robotic table allowing position correction in six degrees of freedom.

### Treatment schedule/follow-up

Treatment was carried out in 5 fractions per week hence over approximately 4 weeks.

The first follow-up examination including clinical examination and diagnostic, contrast-enhanced MRI was carried out 6 weeks post completion of radiation treatment, the second follow-up 3 months thereafter. Further follow-up radiooncological follow-up appointments are scheduled in 6-monthly intervals. The patient was also encouraged to undergo regular check-ups including full ENT clinical examinations in regular intervals (usually every 6 weeks).

## Treatment outcome

**Figure 7:**
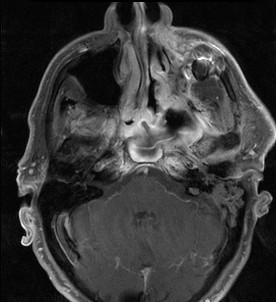
**1st folow-up 6 weeks post completion of RT: complete remission with only posttherapeutic changes: axial, contrast enhanced T1 weighted MRI**.

**Figure 8:**
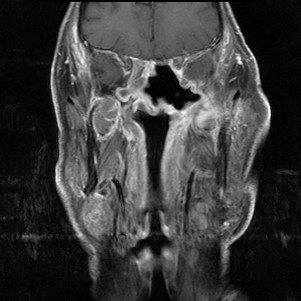
**1st folow-up 6 weeks post completion of RT: complete remission with only posttherapeutic changes: coronal, contrast enhanced T1**.

Treatment was tolerated well. Only very mild skin changes (hyperpigmentation CTC°I and erythema CTC°I, no desquamation) over the left cheek and mild mucous membrane reactions (mucositic CTC °I-II) at the soft/hard left palate could be observed. No change of taste, dysphagia, or weight loss was observed. There was only a very mild xerostomia (CTC°I). When the patient presented to our department again 6 weeks post completion of radiotherapy, he was in a very good clinical state, the pain in the right maxilla was already resolving steadily. While the patient was on oral morphin sulfates during treatment, only needed non-steroidal anti-inflammatories occasionally on follow-up. No residual acute radiogenic reactions could be detected other than residual xerostomia (CTC°I). Furthermore, no residual tumor could be detected on his follow-up MRI-scans 6 weeks post completion of therapy (Figures 7 and 8) and 3 months thereafter.

## Discussion

With the disease being extremely rare, clinicians have to rely on various reported cases for guidance. Hence, the establishment of treatment standards is not possible. Radiotherapy has been controversially discussed in the past. Most reported cases underwent surgical removal.

Reports of ameloblastic carcinomas receiving radiation therapy are scarce and mostly from the pre-3 D and cobalt era [9-11]. To our knowledge, radiotherapy has only been given as adjuvant therapy in only a few cases [11-15] within the past 20 years. Radiation doses between 41,4 Gy and 54 Gy have been comparatively conservative [12,13,16] or not been reported [2,14,17] leading to local relapse in half of the cases. Higher radiation doses between 66 and 72 Gy in close margin/positive-margin resections as reported by Philip et al [15] lead to local control for the duration of available follow-up (0.8 - 3.3 years) in the reported 3 cases.

While it has been discussed in cases with incomplete resections or nodal metastases, there is no evidence for radiotherapy as a potentially definitive treatment modality yet. Faced with the opinion that aggressive treatment (recommending surgical wide excision with 2-3 cm margins [1]) is warranted to counterbalance high tendency of local relapse, RT was given in a high-precision technique as carbon ion therapy. Carbon ion therapy in active beam application with raster-scanned particle beams is able to produce extremely steep gradients hence delivering high doses to the tumor while sparing normal surrounding tissues. In contrast to intensity-modulated radiation therapy, integral dose to the irradiated volume is substantially lower. Also, increased biological effectiveness of carbon ion beams has been shown to be beneficial in other radioresistant tumors [18-20].

While it is beyond the scope of this case report to establish a clinical standard, our case shows that fast complete remissions of extensive ameloblastic carcinomas are possible using carbon ion therapy at substantial doses. Moreover, this treatment is accompanied by very mild treatment-related side effects (erythema, xerostomia CTC°I and mucositis CTC °I-II) and no major radiation-related toxicity; hence the patient could be spared extensive, mutilating and potentially incomplete surgical procedures.

To our knowledge, this is the first case of ameloblastic carcinoma being treated with carbon ion therapy and resulted so far in an excellent posttherapeutic outcome. Therefore radiotherapy with carbon ions should be considered in the definitive treatment of these rare tumors.

## Conflicts of interest

The authors declare that they have no competing interests.

## Authors' contributions

All authors read and approved the final manuscript
